# Polo-like kinase in trypanosomes: an odd member out of the Polo family

**DOI:** 10.1098/rsob.200189

**Published:** 2020-10-14

**Authors:** Yasuhiro Kurasawa, Tai An, Ziyin Li

**Affiliations:** Department of Microbiology and Molecular Genetics, McGovern Medical School, University of Texas Health Science Center at Houston, Houston, TX 77030, USA

**Keywords:** *Trypanosoma brucei*, Polo-like kinase, flagellum, cytokinesis

## Abstract

Polo-like kinases (Plks) are evolutionarily conserved serine/threonine protein kinases playing crucial roles during multiple stages of mitosis and cytokinesis in yeast and animals. Plks are characterized by a unique Polo-box domain, which plays regulatory roles in controlling Plk activation, interacting with substrates and targeting Plk to specific subcellular locations. Plk activity and protein abundance are subject to temporal and spatial control through transcription, phosphorylation and proteolysis. In the early branching protists, Plk orthologues are present in some taxa, such as kinetoplastids and *Giardia*, but are lost in apicomplexans, such as *Plasmodium*. Works from characterizing a Plk orthologue in *Trypanosoma brucei*, a kinetoplastid protozoan, discover its essential roles in regulating the inheritance of flagellum-associated cytoskeleton and the initiation of cytokinesis, but not any stage of mitosis. These studies reveal evolutionarily conserved and species-specific features in the control of Plk activation, substrate recognition and protein abundance, and suggest the divergence of Plk function and regulation for specialized needs in this flagellated unicellular eukaryote.

## 1. Introduction

**Figure 1.**
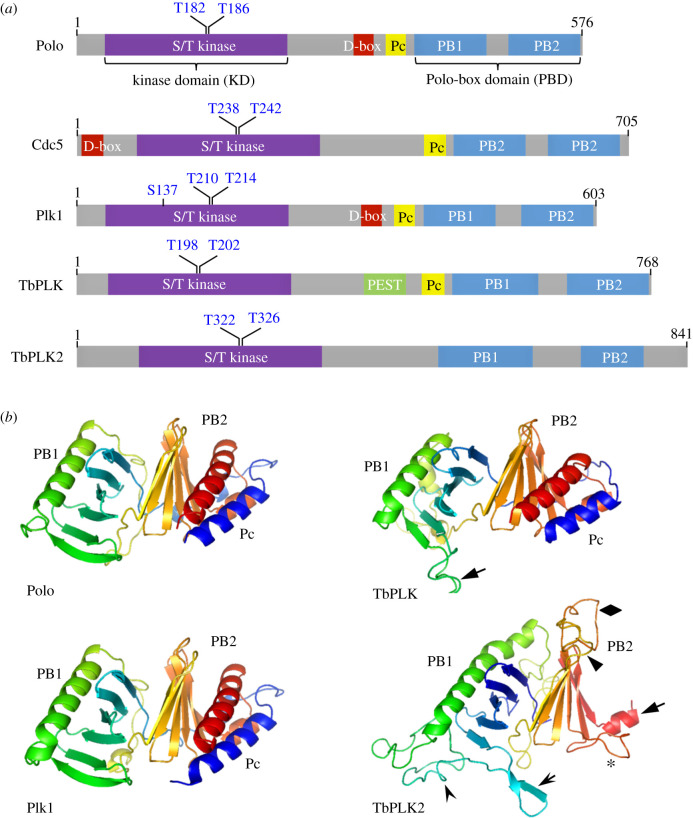
The Polo kinase orthologues in diverse organisms. (*a*) Domain structure and key residues for kinase activation of the Polo kinase family from *D. melanogaster* (Polo), *S. cerevisiae* (Cdc5), *Homo sapiens* (Plk1) and *T. brucei* (TbPLK1 and TbPLK2). PB, Polo box; Pc, Polo-box cap; D-box, destruction box; PEST, Proline-, Glutamate-, Serine- and Threonine-enriched sequence. (*b*) Structural modelling of the PBD of the Plks from *Drosophila*, humans and *T. brucei* using the SWISS-MODEL Server (https://swissmodel.expasy.org/). The following PDB templates were used for modelling. 1UMW for Polo; 6GY2 for Plk1; 4J7B for TbPLK; 5J19 for TbPLK2.

The Polo kinase was originally discovered in *Drosophila melanogaster* more than 30 years ago and named after the phenotype of abnormal spindle poles in a mitotic mutant [1,2]. It is an evolutionarily conserved serine/threonine protein kinase characterized by an N-terminal serine/threonine kinase domain (KD) and a C-terminal Polo-box domain (PBD) consisting of two polo boxes (PB1 and PB2) (figure 1*a*). Polo kinase and its orthologues in other eukaryotic organisms have been established as central regulators of the cell cycle, playing crucial roles in various stages of mitosis and cytokinesis [3,4]. Close orthologues of Polo kinase are found in many eukaryotic species, but not higher plants, such as *Arabidopsis thaliana*, and apicomplexan parasites, such as *Plasmodium*. Plk orthologues are also present in the early divergent kinetoplastid parasites, including *Trypanosoma brucei*, but the functions and regulations of the essential *T. brucei* Plk orthologue (TbPLK) appear to be strikingly different from the Plk orthologues in other organisms. Notably, TbPLK does not localize to any mitotic structure, and no essential role for TbPLK in mitosis has been uncovered by genetic ablation through RNAi-mediated gene knockdown or by inhibition of TbPLK activity through potent Polo-like kinase inhibitors. In this review, we summarize the unusual features of TbPLK and provide perspectives for future research towards the understanding of its mechanistic functions.

## 2. Polo-like kinase: a master orchestrator of mitosis and cytokinesis in eukaryotes

The eukaryotic organisms with increased complexity of the cell cycle usually contain more Polo-like kinase (Plk) paralogues. The unicellular fungi, such as budding yeast (*Saccharomyces cerevisiae*) and fission yeast (*Schizosaccaromyces pombe*), have a single Plk named Cdc5 and Plo1, respectively, whereas the multicellular metazoans have a minimum of two Plk paralogues with distinct functions. *Drosophila* has another Plk named SAK (or Plk4), which is involved in centriole duplication [5]. Vertebrates have a total of five Plk paralogues, which play distinct functions. In humans, Plk1 is a functional homologue of Polo kinase, playing roles in mitosis and cytokinesis [6], and Plk4 is a functional orthologue of SAK, playing a role in centriole duplication [7]. Other Plk paralogues (Plk2, Plk3 and Plk5) belong to the Plk2 subfamily and are only found in some bilaterian animals [8]. Plk2 is concentrated in centrosomes and required for centriole duplication [9], and Plk3 is required for the G1/S cell cycle transition by promoting cyclin E accumulation and Cdc25A activation for DNA replication [10,11]. Plk5 was discovered as a pseudogene encoding a truncated protein lacking most of the kinase domain, and overexpression of Plk5 arrests cells at a G0/G1-like stage [12].

Polo, Plk1 (in mammals), Cdc5 and Plo1 are all close orthologues that play essential roles in mitosis and cytokinesis [4]. They localize to various mitotic and cytokinetic structures in a species-specific manner. In *Drosophila*, Polo localizes to interphase microtubules, mitotic centrosomes, centromeres, kinetochores, the spindle midzone in anaphase and the midbody during cytokinesis. In mammals, Plk1 has similar localizations as Polo in *Drosophila*, except that Plk1 does not localize to interphase microtubules. In budding yeast, Cdc5 diffuses to the nucleus after G1 phase of the cell cycle and is concentrated in the spindle pole bodies throughout the cell cycle and at the mother bud neck from mitosis to cytokinesis. In fission yeast, Plo1 localizes to the spindle pole bodies and the spindle during mitosis and to the future site of cytokinesis during anaphase [4]. In line with their subcellular localizations, Polo and its close orthologues regulate centrosome maturation, mitotic entry, chromosome segregation, mitotic exit and cytokinesis. Details about the subcellular localizations and molecular functions of Plks have been thoroughly summarized previously [4,6].

## 3. Polo-like kinases in *Trypanosoma brucei* are divergent members of the Polo kinase family

*Trypanosoma brucei*, a flagellated unicellular protozoan and the causative agent of human sleeping sickness in sub-Saharan Africa, belongs to the Excavata supergroup of eukaryotes [13] and has a complex life cycle by alternating between the insect vector tsetse fly and the mammalian host. *Trypanosoma brucei* possesses many unusual features in cell cycle control [14–18]. *Trypanosoma brucei* has a closed mitosis and does not appear to assemble centrioles at the spindle poles during mitosis [19]. The parasite assembles unusual kinetochores composed of highly divergent proteins [20–23] and appears to lack some key cell cycle checkpoint machineries, including the mitotic spindle assembly checkpoint and the mitosis-cytokinesis checkpoint [14,17,24]. At the molecular level, *T. brucei* contains an expanded repertoire of the cyclin–CDK system, but lacks some of the evolutionarily conserved cell cycle regulators, including the actomyosin contractile ring, the cytokinetic machinery found in yeast and animals [14,16,17]. These unusual features of cell cycle control highlight some unique mechanisms of mitosis and cytokinesis, which might be exploited as potential drug targets.

The *T. brucei* genome encodes two Plk paralogues [25], which are also found in other trypanosomatids, including *Trypanosoma cruzi*. In other kinetoplastid parasites, including *Leishmania* spp.*,* however, only one Plk orthologue was found. Both Plks in *T. brucei*, named TbPLK (Tb927.7.6310) and TbPLK2 (Tb927. 6.5100), have characteristic features of Plk, containing an N-terminal KD and a C-terminal PBD consisting of two polo boxes (PB1 and PB2) (figure 1*a*). The two polo boxes of *T. brucei* Plks exhibit similar structure, which was predicted by SWISS-MODEL [26], and each of the two polo boxes consists of six β-sheets (β-sheet no. 1−β-sheet no. 6 in PB1 and β-sheet no. 7−β-sheet no. 12 in PB2) and an α-helix (figure 1*b*). TbPLK, as well as Plk1 and its close orthologues in yeast and animals (but not TbPLK2), contains a Polo-box cap (Pc), an α-helical structure that might form part of PBD (figure 1*a,b*). An insertion of disordered sequence between β-sheet no. 5 and β-sheet no. 6 in PB1 (solid arrow) is found in the PDB of TbPLK, but the overall structure of the PBD of TbPLK is similar to that of Plk1 and its orthologues (figure 1*b*). The PBD of TbPLK2 appears to be somewhat different (figure 1*b*). The α-helix of PB2 (solid arrow) appears to be only half of the size of that in other Plk orthologues (figure 1*b*). Additionally, β-sheet no. 6 of PB1 (open arrow) is split into two parts and extends out towards the PB2, and insertions of disordered sequences between β-sheet no. 6 and the α-helix in PB1 (open arrowhead), between β-sheet no. 7 and β-sheet no. 8 in PB2 (solid arrowhead), between β-sheet no. 9 and β-sheet no. 10 in PB2 (asterisk) and between β-sheet no. 10 and β-sheet no. 11 in PB2 (diamond) are found (figure 1*b*). Given these insertions between the β-sheets and the deletion of the α-helix in PB2, it is unclear whether the PBD in TbPLK2 has the same function as the PBD in other Plk orthologues.

**Figure 2.**
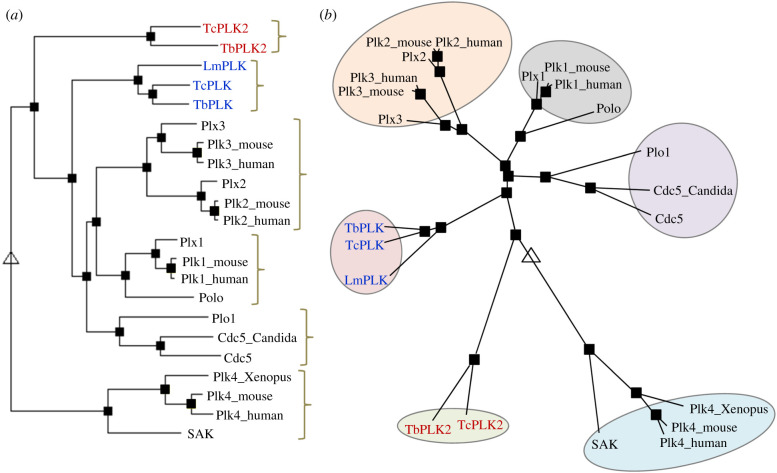
Phylogenetic analysis of Plk family members in diverse organisms. (*a*) Full-length amino acid sequences of known Plk family members from different organisms were aligned using hierarchical clustering, and a rooted tree was constructed based on the sequence alignment obtained using the MultAlin server (http://multalin.toulouse.inra.fr/multalin/). Plks from the following organisms were used: *H. sapiens* (Plk1_human−Plk4_human; accession nos: P53350, Q9NYY3, Q9H4B4 and O00444); *Mus musculus* (Plk1_mouse−Plk4_mouse; accession nos: Q07832, P53351, Q60806 and Q64702); *Xenopus laevis* (Plx1−Plx3 and Plk4_Xenopus; accession nos: P70032, Q90XS4, Q90XS3 and Q6PAD2); *D. melanogaster* (Polo and SAK; accession nos: P52304 and O97143); *Sacchromyces cerevisiae* (Cdc5; accession no.: P32562); *Schizosacchromyces pombe* (Plo1; accession no.: P50528); *Candida albicans* (Cdc5_Candida; accession no.: Q5ABG0); *T. brucei* (TbPLK and TbPLK2; accession nos: Tb927.7.6310 and Tb927.6.5100); *Trypanosoma cruzi* (TcPLK and TcPLK2; accession nos: TcCLB.506513.160 and TcCLB.506945.350); *L. major* (LmPLK; accession no.: LmjF.17.0790). (*b*) A rooted radial tree shows the distance relationship among the Plks from different organisms. The tree was generated using the same hierarchical clustering as in (*a*). The triangle indicates the root of the tree, and the squares indicate branch points.

Phylogenetic analysis of the Plk orthologues from three kinetoplastid parasites, *T. brucei* (TbPLK and TbPLK2), *T. cruzi* (TcPLK and TcPLK2) and *Leishmania major* (LmPLK), and other organisms was performed based on hierarchical clustering [27]. This analysis grouped the Plk orthologues from these kinetoplastid parasites into two distinct clades, the TbPLK/TcPLK/LmPLK clade and the TbPLK2/TcPLK2 clade, and identified the yeast Plk clade (Cdc5/Plo1/Cdc5_Candida) as the closest (figure 2*a,b*). It appears that the TbPLK2/TcPLK2 clade is among the first to diverge from the ancestral Plk, followed by the TbPLK/TcPLK/LmPLK clade, then the Plk orthologues from fungi, and finally the animal Plk1 orthologues (figure 2*b*). These results are consistent with the deep branching position of the kinetoplastid parasites during evolution and suggest that Plk orthologues from kinetoplastid parasites might possess features of the ancestral Plk. These analyses also suggest that kinetoplastid Plks might be functional homologues of Plk1 and its close orthologues in other organisms, such as Cdc5 and Plo1 in fungi and Polo in *Drosophila*.

Functional studies have been carried out for TbPLK, which localize TbPLK to multiple flagellum-associated cytoskeletal structures and the future site of cytokinesis initiation and demonstrate the essentiality of TbPLK in controlling the inheritance of the flagellum-associated cytoskeleton and cytokinesis [28–32]. TbPLK2 has not been extensively characterized, but the work from a genome-wide RNAi screen showed that TbPLK2 was not essential for trypanosome cell viability [33], suggesting that TbPLK2 is not essential under normal growth conditions. TbPLK is probably the only functional Plk orthologue for *T. brucei* proliferation under normal growth conditions; therefore, this review is focused on the unusual features of TbPLK function and regulation.

### 3.1. Polo-like kinase in *T. brucei* localizes to multiple flagellum-associated cytoskeletal structures

**Figure 3.**
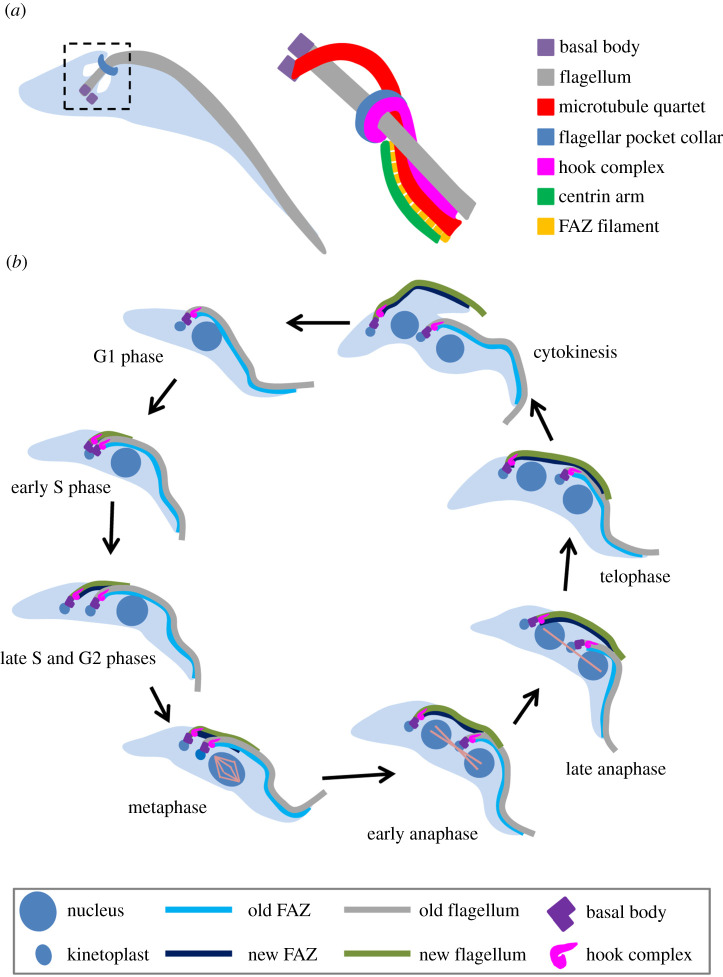
Organization and duplication of flagellum-associated cytoskeletal structures during the cell cycle in *T. brucei*. (*a*) Schematic illustration of the flagellum and flagellum-associated cytoskeletal structures surrounding the proximal portion of the *T. brucei* flagellum. Shown on the left is a *T. brucei* cell at the G1 phase of the cell cycle, which contains a single flagellum. (*b*) Duplication and segregation of flagellum and flagellum-associated cytoskeletal structures during the cell cycle in the procyclic form of *T. brucei*.

A *T. brucei* cell possesses a single motile flagellum, which is originated from the basal body located at the posterior portion of the cell, exits the cell body through the flagellar pocket and extends towards the cell anterior [34] (figure 3*a*). At the exit point of the flagellum, a horseshoe-like cytoskeletal structure termed the flagellar pocket collar (FPC), which is defined by the ring structure-forming protein TbBILBO1 [35], surrounds the flagellum and attaches to the subpellicular microtubule cytoskeleton [36]. At the proximal end of the flagellum between the mature- and pro-basal bodies, a bundle of four specialized microtubules termed the microtubule quartet (MtQ) is assembled, which traverses the FPC and then inserts into the subpellicular microtubule corset (figure 3*a*). Sitting atop the FPC is another cytoskeletal structure termed the hook complex, which is defined by TbMORN1 [37] and TbLRRP1 [38]. The shank part of the hook complex associates with a centrin-marked structure named centrin arm and, in between the hook shank and the centrin arm, lays a filamentous cytoskeletal structure termed the flagellum attachment zone (FAZ), which originates from the hook complex region and extends towards the distal tip of the cell body [39] (figure 3*a*). The FAZ is necessary for flagellum attachment to the cell body [40,41], and the length of the FAZ and the flagellum determines the cell division plane [41,42].

The flagellum and its associated cytoskeletal structures, including the basal body, the FPC, the hook complex and the FAZ, are duplicated and segregated during the cell cycle (figure 3*b*). During the S phase of the cell cycle, the basal body is the first of these cytoskeletal structures to duplicate, with the pre-existing probasal body matured and two new probasal bodies assembled adjacent to the two mature basal bodies. A new flagellum then is assembled from the newly matured basal body and, through an anti-clockwise rotational movement of the new basal body around the old flagellum [36], the new flagellum and its associated new basal body are positioned to the posterior of the old flagellum and its associated old basal body. In the meantime, other flagellum-associated cytoskeletal structures, such as the hook complex, the FAZ and the FPC, are also duplicated, and the newly formed cytoskeletal structures move along with the new flagellum to the posterior of the old flagellum and its associated cytoskeletal structures. The distal tip of the new flagellum is tethered to the lateral side of the old flagellum through a specialized structure termed flagella connector, a mobile transmembrane junction [43,44]. Following cell cycle progression to G2 phase and mitosis, the newly assembled flagellum and FAZ elongate and extend towards the cell anterior, whereas the newly formed hook complex and the FPC retain their original locations at the proximal region of the newly assembled flagellum (figure 3*b*). Due to the elongation of the new flagellum, its associated basal body, hook complex and FPC are moved towards the posterior portion of the cell, resulting in the separation of them from the old structures.

**Figure 4.**
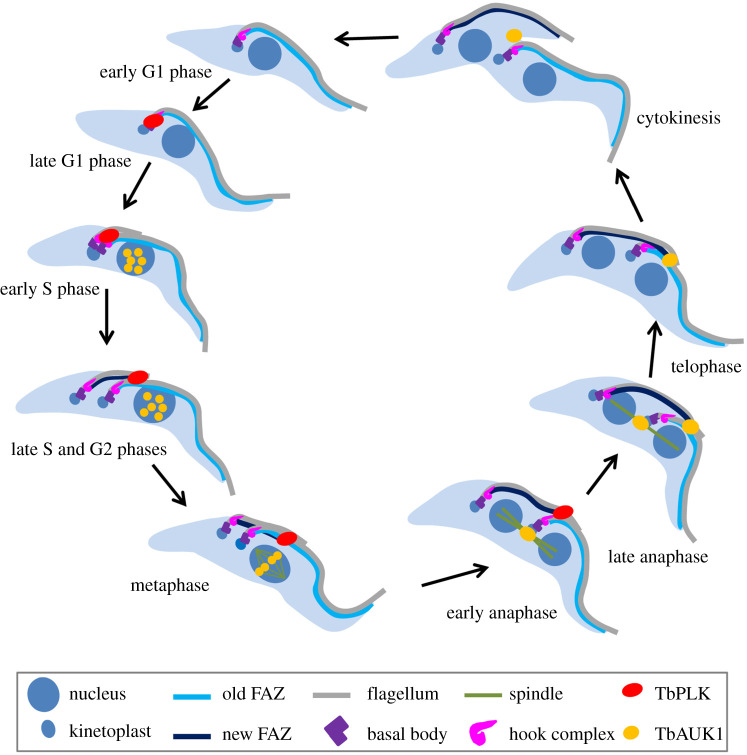
Subcellular localizations of TbPLK and the Aurora B kinase TbAUK1 during the cell cycle in *T. brucei*. Shown are the localizations of the two protein kinases in the procyclic form of *T. brucei*. Note that TbPLK and TbAUK1 do not co-localize at any stage of the cell cycle.

The essential Plk orthologue in *T. brucei*, TbPLK, does not localize to any mitotic structures throughout the cell cycle in the procyclic (insect) form of the parasite [28–30]. Instead, TbPLK localizes to multiple flagellum-associated cytoskeletal structures, including the basal body, the hook complex, the flagella connector and the distal tip of the newly assembled FAZ in the insect form [30–32,45–47]. TbPLK is not detectable at any subcellular structures at the early G1 phase of the cell cycle, but it appears at the basal body and the hook complex during late G1 phase (figure 4). At early S phase when the new FAZ starts to form from the hook complex region, TbPLK emerges at the newly assembled FAZ. At late S phase, TbPLK remains at the distal tip of the elongating new FAZ, but it disappears from the basal body and the hook complex. Following cell cycle progression from G2 phase to early anaphase, TbPLK remains associated with the distal tip of the new FAZ, and it disappears from the new FAZ tip from late anaphase onward (figure 4). The dynamic localization of TbPLK at various flagellum-associated cytoskeletal structures during the cell cycle suggests its roles in regulating the biogenesis or segregation of these cytoskeletal structures.

### 3.2. Polo-like kinase in *T. brucei* controls the inheritance of flagellum-associated cytoskeletal structures

The physiological function of TbPLK has been investigated by RNAi-mediated ablation in both the procyclic and bloodstream forms of the *T. brucei* life cycle. However, the mechanistic roles of TbPLK are only elucidated in the procyclic form of *T. brucei*. Thus, this review will focus on the findings discovered in the procyclic form. Consistent with the finding that TbPLK is not detectable in any mitotic structures, knockdown of TbPLK does not inhibit mitosis [28–30]. This is in striking contrast to the essential role of Plk1 and its close orthologues from yeast and animals in regulating multiple stages of mitosis (see above). TbPLK contains a nuclear localization sequence in the inter-domain linker between the KD and the PBD, which is capable of targeting TbPLK to the nucleus only when the PBD is deleted [32]. It suggests that the nuclear localization sequence in TbPLK might be embedded in a position that does not allow access of the nuclear import machinery.

TbPLK appears to play distinct roles in the duplication and segregation of the flagellum-associated cytoskeletal structures to which TbPLK localizes during the cell cycle. TbPLK does not play any role in regulating the biogenesis of the new basal body, but rather controls the segregation of the duplicated mature basal body–probasal body pairs [32,48]. In the basal body, TbPLK phosphorylates a trypanosome-specific protein named SPBB1, which is required for basal body segregation [47]. Therefore, one essential role of TbPLK in the basal body is to regulate certain basal body component(s) to promote basal body segregation. It remains unclear whether TbPLK also regulates other basal body proteins, but it appears that TbPLK does not regulate the basal body cartwheel protein TbSAS-6 [49], in contrast with that in animals where Polo-like kinase 4 regulates SAS-6 to control centriole biogenesis [50]. Unlike its role in controlling basal body segregation, TbPLK plays an essential role in promoting the duplication of the hook complex [30]. At the hook complex, TbPLK phosphorylates TbCentrin2, the centrin2 homologue in *T. brucei* [51] that localizes to the centrin arm structure of the hook complex, and this phosphorylation appears to play a role in hook complex duplication [52]. This finding provides a molecular basis for the role of TbPLK in promoting the duplication of the hook complex.

TbPLK is also required for the assembly of the FAZ, as knockdown of TbPLK produces cells with a new FAZ that is significantly shorter than that in wild-type cells [31]. As a consequence of defective FAZ assembly, the newly synthesized flagellum is detached from the cell body [31]. The mechanistic role for TbPLK in promoting FAZ assembly remains unclear. Proper duplication of the hook complex is necessary for FAZ assembly [38,52], and TbPLK depletion disrupts hook complex duplication [30]. Therefore, it is possible that the defect in FAZ assembly caused by TbPLK depletion is attributed, at least in part, to the defective duplication of the hook complex [30,52]. Despite being detached from the cell body, however, the new flagellum is still of normal length and structure, albeit it locates closely to the old flagellum rather than being positioned towards the posterior portion of the cell [31]. The positioning and attachment of the newly assembled flagellum, coined as flagellum inheritance [31], appear to depend on the assembly/elongation of the new FAZ [41,53–56] and the segregation of duplicated basal bodies [47]. TbPLK thus regulates flagellum inheritance through promoting hook complex duplication, basal body separation and FAZ assembly.

### 3.3. Polo-like kinase in *T. brucei* regulates cytokinesis through a trypanosome-specific signalling pathway

A *T. brucei* cell divides through binary fission along its longitudinal axis in a uni-directional manner from the anterior tip of the new-flagellum daughter cell towards the posterior tip of the old-flagellum daughter cell [57–59]. The cell division plane in a dividing *T. brucei* cell is determined by the length of the new flagellum and the new FAZ [41,42], and prior to cytokinesis initiation, a division fold is formed along the cell division plane through membrane invagination between the new flagellum and the old flagellum [58]. Cleavage furrow ingresses from the distal tip of the new-flagellum daughter cell, and proceeds towards the posterior end of the old-flagellum daughter cell [58]. *Trypanosoma brucei* lacks a homologue of the class II myosin motor protein [60], a crucial component of the actomyosin contractile apparatus located at the cleavage furrow in yeast and animals [61]. Moreover, the actin protein in *T. brucei* is involved in endocytosis but not cytokinesis [62]. *Trypanosoma brucei* appears to use distinct mechanisms to control cytokinesis.

TbPLK plays a critical role in promoting cytokinesis initiation by regulating a cohort of trypanosome-specific regulators [47,59,63–71]. This specific function in cytokinesis initiation is probably carried out by TbPLK at the distal tip of the new FAZ [30–32,45,59], as its downstream factors all localize to the new FAZ tip. One of the TbPLK downstream factors is the Aurora B kinase homologue TbAUK1 [72,73], which forms a unique chromosomal passenger complex (CPC) with a highly divergent INCENP homologue TbCPC1 and a trypanosome-specific protein named TbCPC2 [74]. The CPC displays a dynamic subcellular localization by localizing to kinetochores from S phase to metaphase, the central spindle during anaphase, the distal tip of the new FAZ during late anaphase and telophase, and finally the ingressing cleavage furrow during cytokinesis [57,74] (figure 4). Consistent with its localizations to mitotic and cytokinetic structures, the CPC plays multiple roles in spindle assembly, chromosome segregation and cytokinesis [57,72–75]. While the requirement of the CPC for cytokinesis initiation and cytokinesis progression is believed to be attributed to its localization to the new FAZ tip and the cleavage furrow, no evidence has been presented to demonstrate that the CPC executes its function at the cleavage furrow. Nevertheless, when TbAUK1 emerges at the distal tip of the new FAZ during late anaphase, TbPLK has disappeared from the new FAZ tip [76]; therefore, the two protein kinases do not meet with each other throughout the cell cycle (figure 4). The sequential localizations of TbPLK and TbAUK1 to the new FAZ tip suggest their order of action in regulating cytokinesis, and the impairment of TbAUK1 localization to the new FAZ tip by knockdown or inhibition of TbPLK [59] demonstrates that TbPLK acts upstream of TbAUK1 in the cytokinesis signalling pathway.

**Figure 5.**
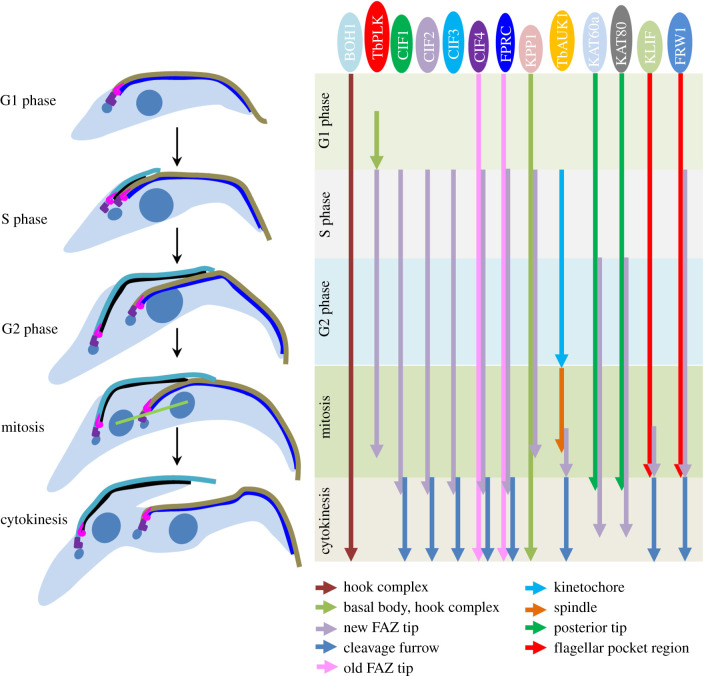
TbPLK and its upstream and downstream factors in the cytokinesis regulatory pathway in *T. brucei*. Shown is the summary of the temporal and spatial localizations of TbPLK and other cytokinesis regulatory proteins during the cell cycle of the procyclic form of *T. brucei*. Coloured arrows indicate different subcellular structures to which the cytokinesis regulators localize from one cell cycle stage (start of the arrow) to another cell cycle stage (end of the arrow).

Subsequent efforts attempted to discover TbPLK-interacting proteins by yeast two-hybrid identifies a trypanosome-specific protein named CIF1 based on its localization to the new FAZ tip and the cleavage furrow and its essential role in cytokinesis initiation [47,59]. CIF1 was also independently identified by proximity-dependent biotin identification with TbPLK as bait and was named TOEFAZ1 based on its localization to the new FAZ tip [63]. However, CIF1 additionally localizes to the cleavage furrow during cytokinesis [59]. During early cell cycle stages from early S phase to early anaphase, CIF1 co-localizes with TbPLK at the new FAZ tip, and during late cell cycle stages from late anaphase to cytokinesis, CIF1 co-localizes with TbAUK1 at the new FAZ tip and the ingressing cleavage furrow [59]. CIF1 interacts with both TbPLK and TbAUK1 and is a substrate of both kinases (Y.K. & Z.L. 2020, unpublished results), and genetic analyses demonstrate that CIF1 functions downstream of TbPLK and upstream of TbAUK1 [59], suggesting that CIF1 bridges TbPLK and TbAUK1 in the cytokinesis signalling pathway. Knockdown of TbPLK or inhibition of TbPLK activity disrupts CIF1 localization to the new FAZ tip [59,68] and, conversely, depletion of CIF1 also impairs TbPLK localization to the new FAZ tip [63]. Subsequent work by BioID using CIF1 as bait and follow-up BioID experiments with the proteins identified by CIF1 BioID discover a cohort of trypanosome-specific proteins, including CIF2, CIF3, CIF4, FPRC, KLIF, KPP1 and FRW1 [64–66,69,70], and an evolutionarily conserved katanin complex, the KAT60a–KAT80 complex [64], that form complexes with CIF1 or function downstream of CIF1. These findings place TbPLK at the upstream of a novel cytokinesis regulatory pathway in *T. brucei*. Prior to localizing to the new FAZ tip at early S phase, TbPLK localizes to the hook complex, where it interacts with a hook complex-associated protein named BOH1 [77]. BOH1 is involved in maintaining the morphology of the hook complex and promoting cytokinesis initiation by targeting TbPLK to the hook complex [77]. Knockdown of BOH1 also disrupts CIF1 localization to the new FAZ tip, presumably through impairing TbPLK localization, as localization of CIF1 requires TbPLK. However, knockdown of TbPLK does not affect BOH1 localization to the hook complex [77], suggesting that BOH1 acts upstream of TbPLK in the cytokinesis regulatory pathway. The spatio-temporal localization of TbPLK and other cytokinesis regulators is summarized in figure 5.

### 3.4. The regulation of TbPLK possesses both evolutionarily conserved and trypanosome-specific features

Like its counterparts in yeast and metazoans, TbPLK is also activated by phosphorylation of a threonine residue, Thr-198, in the activation loop (T-loop) of the kinase domain [32]. Thr-198 in TbPLK is equivalent to Thr-210 in human Plk1 [78], which is phosphorylated by Aurora A kinase at G2 phase to activate Plk1 to promote mitotic entry [79,80]. Phosphorylation of Thr-210 in Plk1 and the equivalent Thr-182 in Polo is also mediated by Aurora B kinase in centromeres and kinetochores, and this phosphorylation is required for mitotic progression [81]. *Trypanosoma brucei* does not have a close homologue of Aurora A kinase [72], but expresses two essential Aurora kinase homologues named TbAUK1 and TbAUK2, which do not co-localize with TbPLK at any stage of the cell cycle [76,82], and a non-essential Aurora kinase homologue named TbAUK3 [72,73], suggesting that none of the three Aurora kinase paralogues is responsible for Thr-198 phosphorylation. TbPLK is also activated by phosphorylation of another threonine residue, Thr-202, in the T-loop [32]. The phosphorylation of the corresponding site in Cdc5, Thr-242, is also required for activation of Cdc5, and this phosphorylation is mediated by the cyclin-dependent kinase Cdc28 in budding yeast [83]. It is possible that phosphorylation of Thr-202 in TbPLK is similarly carried out by a cyclin-dependent kinase, and this might occur in an early cell cycle stage during which TbPLK functions to promote basal body separation and hook complex duplication. The *T. brucei* genome encodes 11 Cdc2-related kinases (CRKs) [14], and it remains to determine which of them is responsible for Thr-202 phosphorylation. In human Plk1, phosphorylation of Ser-137, which is equivalent to Thr-125 in TbPLK, partially activates Plk1 [78,84]. It remains unknown whether Thr-125 in TbPLK is phosphorylated and whether phosphorylation of Thr-125 is required for TbPLK activation. Nonetheless, it appears that activation of Plk through Ser/Thr phosphorylation in the T-loop is conserved across the eukaryotic organisms. In addition to Thr-198 and Thr-202, TbPLK is phosphorylated at four sites, Thr-10, Ser-12, Ser-13 and Ser-14, in the N-terminal tail prior to the KD and at eight sites, Ser-338, Ser-388, Ser-462, Thr-465, Thr-466, Thr-468, Thr-469 and Ser-506, in the inter-domain linker between the KD and the PBD [85,86]. The functions of these phosphosites remain to be explored.

The structural basis for phosphorylation-mediated PLK activation has been elucidated. The phosphorylation of T-loop in Plk1 induces a conformational change to promote the catalytic activity of Plk1 [87]. The phosphorylation of Thr-210 in Plk1 is also thought to lock Plk1 in an open conformation, in which the KD and the PBD are dissociated [88]. Plk1 and its close orthologues are auto-inhibited by intra-molecular interactions between the KD and the PBD, and binding of phosphopeptides to the PBD dissociates the PBD from the KD, thereby leading to the relief of the inhibitory effect exerted by PBD binding. Priming phosphorylation of Plk substrates is generally required for binding the PBD and relieving the auto-inhibition on the KD [89–93] and, in some cases, phosphorylation of a substrate by Plk creates a self-tethering site for its specific binding to the PBD and targets Plk to specific subcellular locations [91]. In human Plk1, phosphorylation of Ser-137, but not Thr-210, dissociates the PBD from the KD, thereby partially activating Plk1 [94]. In *T. brucei* TbPLK, the PBD also binds the KD and inhibits TbPLK kinase activity, but it does not bind certain substrates of TbPLK, such as TbCentrin2 and SPBB1 [32]. Moreover, the PBD of TbPLK does not appear to be involved in targeting TbPLK to specific subcellular locations, and the four conserved residues within the PBD of PLK that are implicated in direct binding to phosphopeptides are all missing in the PBD of TbPLK [32]. It is possible that binding of some TbPLK substrates does not required priming phosphorylation.

The protein abundance of TbPLK is under stringent control during the cell cycle in *T. brucei*. TbPLK in the basal body and the hook complex is degraded after the transition from G1 to S phase by a Cullin-RING ubiquitin ligase (CRL)-mediated degradation machinery, and degradation of TbPLK in these flagellum-associated cytoskeletal structures is required for flagellum inheritance [46]. This specific CRL, CRL4^WDR1^, is composed of Cullin4, DDB1 and a WD40 repeats-containing protein named WDR1, and recognizes the PEST motif, a stretch of sequence rich in proline (P), glutamic acid (E), serine (S) and threonine (T) and known as a signal sequence for protein degradation [95], in the inter-domain linker between the KD and the PBD [46]. Notably, degradation of TbPLK also requires the destruction boxes (D-box) and requires another ubiquitin ligase, the anaphase-promoting complex/cyclosome (APC/C), albeit the PEST motif plays the major role in mediating TbPLK degradation. It is possible that CRL4^WDR1^ mediates TbPLK ubiquitination and degradation in the basal body and the hook complex after the G1/S transition and APC/C mediates TbPLK ubiquitination and degradation in the distal tip of the new FAZ during anaphase. In budding yeast and *Drosophila*, degradation of Cdc5 and Polo also depends on the D-box [96–98]. In humans, degradation of Plk1 requires APC/C and depends on a D-box [96], and degradation of Plk4 requires the Cullin-RING ubiquitin ligase SCF^β-TrCP/Slimb^ [99,100] to recognize the PEST motif that is trans-phosphorylated by Plk4 itself [101,102]. In this regard, TbPLK appears to possess certain properties that are shared by both Plk1 and Plk4 in humans. However, CRL4^WDR1^-mediated degradation of TbPLK does not require phosphorylation of the PEST motif [46], suggesting that the signal for targeting TbPLK for degradation is distinct from the signal for degradation of human Plk4.

## 4. Concluding remarks and perspectives

The essential involvement of TbPLK in regulating the inheritance of multiple flagellum-associated cytoskeletal structures, including the basal body, the hook complex and the FAZ, suggests a divergent function for this evolutionarily conserved protein kinase in a protozoan parasite that relies on the flagellum for locomotion, cell morphogenesis, cell division and cell–cell communication. *Trypanosoma brucei* lacks centriole-like structures at the spindle poles for chromosome segregation, but possesses the centriole-like basal body for nucleation of the flagellum. Duplication and segregation of the basal body are among the earliest events in the cell cycle of *T. brucei*, and the implication of TbPLK in the process of basal body segregation reflects the conserved function of Plks in the duplication and maturation of centrioles in animals and spindle pole bodies in yeast. Given that TbPLK is not detectable in nucleus and plays no essential role in mitosis, it suggests that the function of Plks in regulating multiple mitotic events in yeast and animals might be acquired at a later time during evolution after the centriole/spindle pole body structure was adopted for chromosome segregation.

Since the hook complex is a trypanosome-specific cytoskeletal structure, the requirement of TbPLK for hook complex duplication suggests a divergent function of Plk to meet the special needs in *T. brucei*. The FAZ is a trypanosomatid-specific cytoskeletal structure, and the distal tip of the newly assembled FAZ intracellular filament constitutes the initiation site of cytokinesis in *T. brucei*. Proper assembly/elongation of the FAZ also impacts cell division, as the length of the new FAZ determines the cell division plane. Therefore, the localization of TbPLK to the distal tip of the new FAZ filament and the requirement of TbPLK for cytokinesis initiation reflect the conserved function of Plks in promoting cytokinesis initiation. TbPLK localizes to the new FAZ tip from early S phase and disappears before the completion of mitosis, indicating that TbPLK is localized to the future site of cytokinesis and acts as an upstream regulator in the cytokinesis signalling pathway. Hence, TbPLK probably does not play roles in regulating mitotic exit and the cellular events directly related to cytokinesis. This feature of TbPLK contrasts from that of Plk1 and its close orthologues in yeast and animals, where they are all concentrated at the site of cytokinesis and are directly involved in cytokinesis, despite the assembly of different cytokinesis architectures, such as the septum in fission yeast and the cleavage furrow in budding yeast and animals.

The mechanistic roles of TbPLK in facilitating the inheritance of the flagellum and its associated cytoskeletal structures and promoting the initiation of cytokinesis remain poorly understood. It is believed that TbPLK exerts its function through phosphorylation of certain substrates at multiple subcellular locations, but only a few substrates have been validated and how phosphorylation impacts their functions has not been elucidated, thus hindering the dissection of the underlying mechanisms. Moreover, the mechanism for TbPLK activation is also unclear, as the upstream protein kinase(s) that phosphorylates TbPLK in the T-loop (Thr-198 and Thr-202) has not yet been identified. Further, reversible protein phosphorylation mediated by a protein kinase and an antagonizing protein phosphatase participates in numerous cellular processes in eukaryotes. A kinetoplastid-specific protein phosphatase named KPP1 also regulates flagellum inheritance and cytokinesis initiation [64,69,70], and it partly co-localizes with TbPLK at the basal body, the hook complex and the new FAZ tip [70]. It remains unclear whether KPP1 antagonizes TbPLK function by dephosphorylating TbPLK substrates, as is the case of PP2A-mediated counteraction of Plk1 in humans [103]. It is also unclear whether KPP1 antagonizes TbPLK function by dephosphorylating Thr-198 and/or Thr-202 of TbPLK to inactivate TbPLK, as is the case of PP1Cβ-mediated counteraction of Plk1 in humans [104]. Finally, the PBD of TbPLK lacks all of the conserved residues involved in binding phosphopeptides, does not associate with known TbPLK substrates, and is unable to target TbPLK to its subcellular locations [32]. TbPLK expressed ectopically in HeLa cells fails to localize to mitotic and cytokinetic structures, and Plk1 expressed ectopically in *T. brucei* fails to localize to flagellum-associated cytoskeletal structures [32], suggesting that the PBDs from TbPLK and Plk1 are not inter-exchangeable. These findings raise questions of whether the PBD has distinct functions in *T. brucei* and how TbPLK localization is regulated. Future efforts directed to identify and characterize TbPLK substrates and dissect the function of the PBD will help to delineate the signalling cascades controlling the inheritance of flagellum and flagellum-associated structures and the initiation of cytokinesis and to understand the mechanism underlying TbPLK regulation.

## Data accessibility

This article has no additional data.
